# Easier detection of invertebrate "identification-key characters" with light of different wavelengths

**DOI:** 10.1186/1742-9994-8-27

**Published:** 2011-10-31

**Authors:** Marcel HM Koken, Jacques Grall

**Affiliations:** 1 CNRS, Castel Nevez 29830 Ploudalmezeau, France; 2 CNRS UMS3113, Observatoire IUEM, UBO, Rue Dumont d'Urville 29280 Plouzané, France

**Keywords:** fluorescent colours, light emission, determination, cryptic species, dimorphism, pattern UV light, deep-blue light, autofluorescence

## Abstract

The marine α-taxonomist often encounters two problems. Firstly, the "environmental dirt" that is frequently present on the specimens and secondly the difficulty in distinguishing key-features due to the uniform colours which fixed animals often adopt.

Here we show that illuminating animals with deep-blue or ultraviolet light instead of the normal white-light abrogates both difficulties; dirt disappears and important details become clearly visible. This light regime has also two other advantages. It allows easy detection of very small, normally invisible, animals (0.1 μm range). And as these light wavelengths can induce fluorescence, new identification markers may be discovered by this approach.

## Introduction

When trying to identify small marine animals the α-taxonomist looks for features that objectively distinguish one species from another, and often he encounters two problems. Firstly, the "dirt" omnipresent in the marine environment sticks onto the specimens thereby often masking hairs, chaetae, bristles, spicules, plates, and the many other features, which require to be observed. Secondly, animals that are fixed in formalin or alcohol show a rather uniform colour (for some this is also true in their native state). Both problems make identification very time consuming and if these could be avoided determination would be easier and quicker for both taxonomists and routine observers. Here we show that illumination with deep blue or UV light completely eliminates above problems.

## Results

We use a Zeiss LUMAR dissecting microscope to screen for fluorescent patterns on marine animals, to decipher potential UV light-induced behavioural signs [1-4] and to identify sources for the isolation of new fluorescent proteins, which may lead to new *in situ *imaging tools [5]. During these screens it became rapidly clear that the use of different light colours has four advantages for the taxonomist:

### Dirt removal

**Figure 1:**
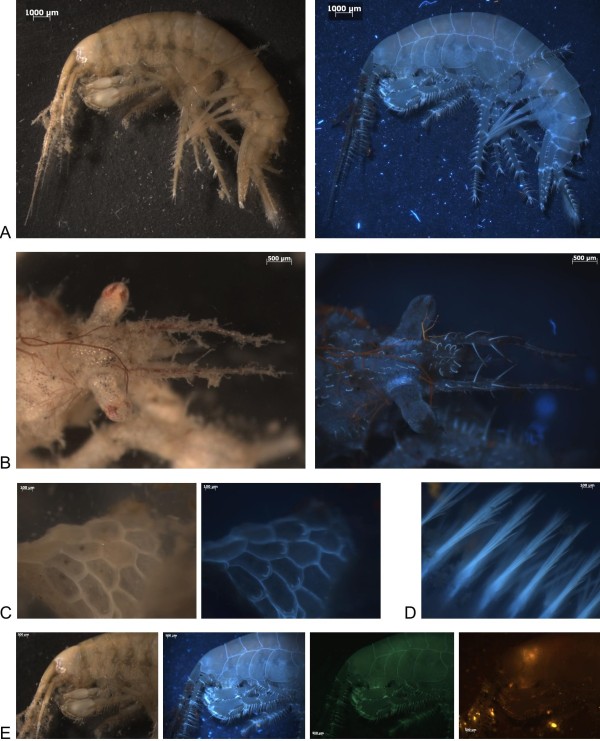
**Use of different light colours permits easier "identification-marker" visualization and "removes dirt"**. A. Unfixed *Gamarus salinus *(Spooner, 1947). Left: "white light", Right: UV light. B. Unfixed *Macropodia linaresi *(Forest & Alvarez, 1964). Left: "white light" Right: UV light. C. Unfixed *Membranipora membranacea (Lineaus, 1767) Left: *"white light"; Middle: UV light. D. Chaetae of a polychaete under UV light. E. Illumination with different light colors of *Gamarus salinus*. Left panel: white light. Left-middle panel: UV light. Right-middle panel: Blue light. Right panel: Green light.

Figure 1 shows that the small amphipod, *Gammarus salina *(Figure 1A, left) and the small crab, *Macropodia linaresi *(Figure 1B, left) are both covered with "dirt" which is clearly visible when exposed to normal light. Note how difficult it is to count the hairs on the crab or to see the hairs or the form of the chitin plates on the amphipod's body. Illumination with UV or with deep blue light results in an "optical" removal of the dirt (Figure 1A, B, C or E, UV panels).

### Easier "identification-marker" visualisation

UV illumination has a second positive effect as it makes plates and hairs on the exoskeleton become more clearly visible (Figure 1A, B, D, E). The animal's exoskeleton seems to "conduct the light", perhaps in a similar way to glass fibres. Note that green light failed to make the exoskeleton "light up", and that in that case dirt continued to remain visible (Figure 1E (right panel)).

As well as in crustaceans, illumination with UV (or deep-blue) light facilitates or ameliorates observation in other animal groups, as is illustrated by worm chaetae (Figure 1D) or by the clearly visible operculum on the bryozoan, *Membranipora membranacea *(Figure 1C).

Both, "dirt removal" and "light conveyance" work well on fresh, frozen (but subsequently thawed), ethanol- or buffered-formalin-fixed specimens (not shown).

### Detection of "invisible" animals

**Figure 2:**
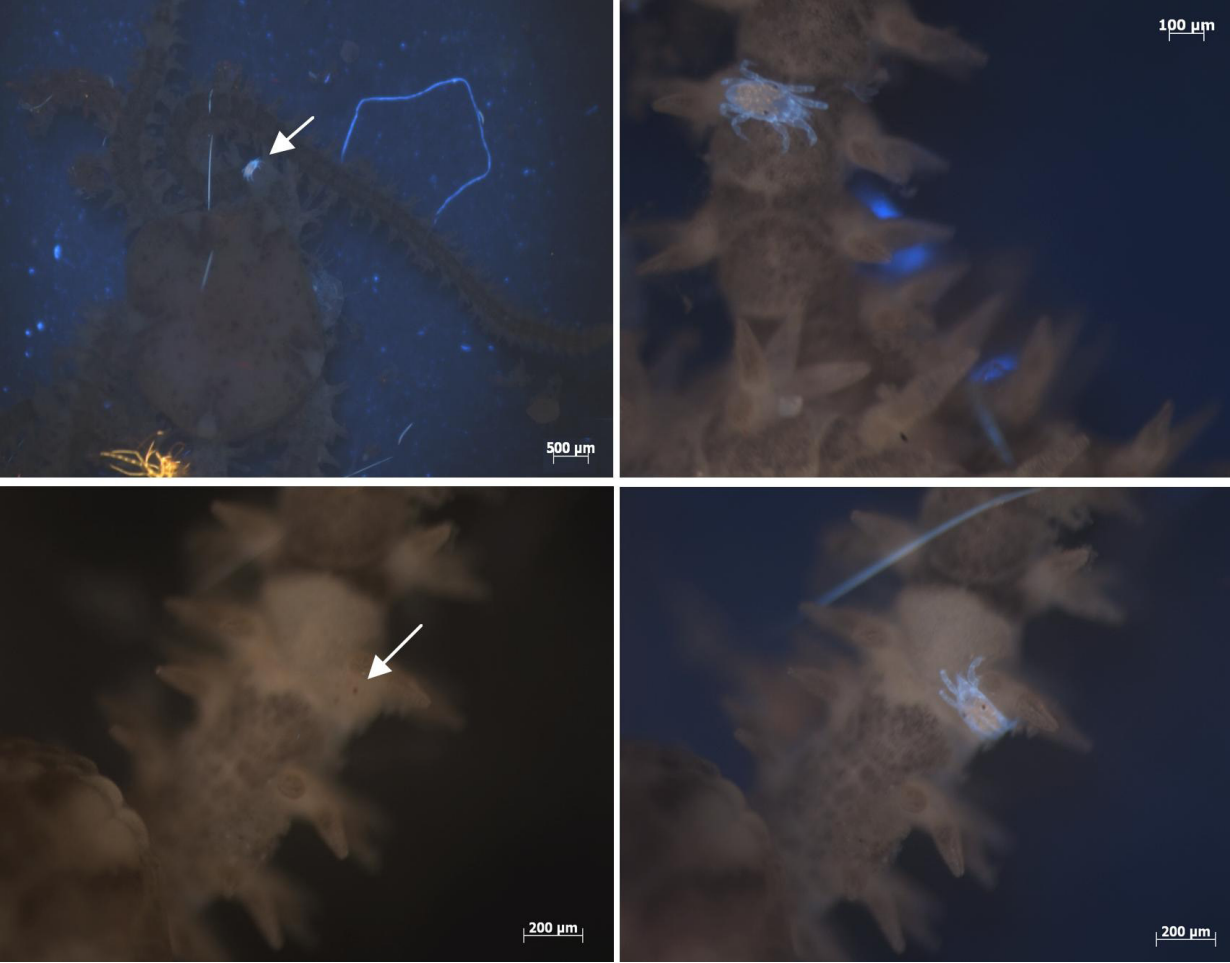
**Different light colours allow visualization of otherwise "invisible" animals**. A (Upper left panel). Illumination of an unfixed brittlestar with UV light. Please note the small mite indicated by the arrow. B (Upper right panel): Zoom of A. C (Lower left panel) and D (lower right panel). An arm of the brittlestar under white (left) and UV light (right). Please note, the two brown eyes (arrow), the only visible part of the mite under white light.

During a routine fluorescence screen, a small brittlestar was inspected under UV light (Figure 2A), and a tiny (200 μm) mite was caught walking over the arms of the echinoderm (Figure 2A (arrow), Figure 2B).

Whereas UV light allowed easy detection of this mite through the "light conveyance phenomenon" (Figure 2D), the animal was invisible without UV illumination; only its brown eyes could be seen (Figure 2C, *arrow*) if one knew where to look.

### Fluorescent identification-markers

**Figure 3:**
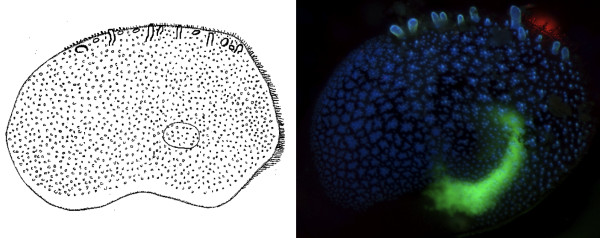
**Different light colours may provide additional identification-markers though the induction of fluorescent patterns**. Left panel. Line drawing of a scale from *Harmothoe impar (Johnston, 1839)*. Right panel. Superposition of UV light and deep-blue light exposure of a scale of *Harmothoe impar*. Note, the blue and green fluorescence that is induced, and the composite form of the "spots" which is invisible with "white light".

Applying different wavelength light/colours has another advantage over the use of white-light as it can induce fluorescence colours on the specimen. Many marine species show specific fluorescent patterns. For instance, in Figure 3 the fluorescence pattern of a scale (elytrum) of the polynoid worm, *Harmothoe impar *shows that a lot of additional information can be obtained which is not visible under white-light illumination. Unfortunately, in most cases this works only on fresh or frozen (and thawed) samples. Fixation rapidly destroys most of the fluors that we have encountered thus far.

## Discussion

We show here that changing the classical white-light illumination for a deep-blue or UV light source has several advantages. The "dirt removal" combined with the "light conveyance" phenomenon speeds up and eases significantly the visualisation of identification markers. It may be possible that in certain cases this approach would avoid the need for staining, or even the use of immunological approaches that are often necessary for revealing certain details. And as even the tiniest of hairs "light up" it may be that scanning electron microscopy could prove sometimes unnecessary for identification.

"Dirt" is generally not a problem when examining terrestrial animals, but the "light conveyance phenomenon" is also a great help for revealing fine details on the bodies of land invertebrates (data not shown).

An additional advantage of both phenomena is that they will facilitate the scientific drawing process. For instance, the picture of Figure 1B (right panel) can very easily be transformed into a simple line drawing with Photoshop or an equivalent program.

To the best of our knowledge both the "dirt removal" and the "light conveyance" phenomena were never reported before. Both are extremely convenient when studying invertebrates as identification markers are much easier visualized with only a change of light colour. The same "light conveyance" phenomenon facilitates also the visualization of small organisms that under normal light regimes remain invisible. We cannot exclude that what we call "light conveyance phenomenon" is in reality the fluorescence of a resistant fluor that is very commonly found in all groups we investigated thus far, and which has a very large excitation and emission spectrum. Although we cannot be sure about the physical explanation of the phenomenon, its usefulness for the taxonomist remains without doubt.

The use of blue light-elicited autofluorescence in medicine started only a few years ago and was found to be very helpful for distinguishing between normal and abnormal or cancerous tissues [6-9]. Autofluorescence depends on the presence of endogenous fluors that are of changing type and which differ in concentrations between various tissues [6,8]. These differences allow therefore visualizing tissue morphological or physiological changes or tissue invasion [6]. By these means the MD can repair or remove the "abnormal" tissues while leaving the healthy tissues unharmed.

In a few reports, autofluorescence is also used to distinguish between closely resembling spores [10-12] or pollen [13]. It can also help by distinguishing between live and dead cells [14] or by allowing the detection of insect larvae which contaminate food [15].

In our screens we often detect specific fluorescence patterns on the animals' bodies and these may provide new identification-markers for correct or if not, easier identification.

These additional fluorescence patterns may allow distinction between similar species [16] or lead to the identification of thus far invisible sexual dimorphism or patterns that change during development.

These patterns are presently ignored by the animal taxonomist, but should not be excluded as they may represent additional valid identification markers

In conclusion, we believe that inspecting specimens with different light colours, most importantly with UV or deep blue light, will greatly facilitate the identification of specimens. Moreover, this approach has the potential to detect (sub)species, dimorphisms or developmental patterns, which under normal light regimes remain completely invisible and may be still unknown to science.

## Methods

### Samples

Animals used in this study were collected at low tide at "Melon-Porspoder" and "Le Dellec - Plouzané" (Brittany, France). Moving live animals were narcotized, mostly by immersion in a 7.5% MgCl_2 _solution in seawater, or by other described methods [17]. All biological material has been collected under appropriate collection permits and approved ethics guidelines.

### Fixation conditions

Animals were examined either after fixation or in native condition. Fixation was done in 70% ethanol (in seawater) for 1 hour or for 4 days, or in 7% buffered formalin (in seawater) for 1 hour or 4 days. After fixation specimens were transferred into sterile seawater for long-term storage.

### Microscopy

Samples were inspected on a Zeiss LUMAR V12 dissecting microscope with a Axiocam MrC5 colour camera 5. The following excitation/emission filter sets were mounted:

- set 01 UV, Ex BP 365/12, EM LP 397 ("UV" in the figure legends).

- set 38 GFP, Ex BP 470/40, EM BP 525/50 ("blue").

- set 43 Cy3, Ex BP 545/25, EM BP 605/70 ("green").

## Abbreviations

UV: ultraviolet light; MD: medical doctor.

## Competing interests

The authors declare that they have no competing interests.

## Authors' contributions

Both authors contributed equally to this work. Both authors read and approved the final manuscript.
